# Objective Versus Subjective Effort in Schizophrenia

**DOI:** 10.3389/fpsyg.2020.01469

**Published:** 2020-07-09

**Authors:** Isabel Kreis, Steffen Moritz, Gerit Pfuhl

**Affiliations:** ^1^Department of Psychology, Faculty of Health Sciences, UiT – The Arctic University of Norway, Tromsø, Norway; ^2^Department of Psychiatry and Psychotherapy, University Medical Center Hamburg-Eppendorf, Hamburg, Germany

**Keywords:** digit span, mental effort, task load, motivation, schizophrenia, pupillometry

## Abstract

**Background and Objectives:**

Performance on cognitive tasks is often impaired in individuals with schizophrenia (SCZ), possibly resulting from either cognitive deficits (e.g., limited working memory capacity) or diminished mental effort or both. Investment of mental effort itself can be affected by cognitive resources, task load, and motivational factors and has thus proven difficult to measure. Pupil dilation during task performance has been proposed as an objective measure, but it remains unclear to what extent this converges with self-reports of perceived task demands, motivation, and invested effort. The current study tried to elucidate this question.

**Methods:**

A visual version of the digit span task was administered in a sample of 29 individuals with a diagnosis from the SCZ spectrum and 30 individuals without any psychiatric disorder. Pupil size was recorded during the task, whereas self-reported invested effort and task demand were measured afterward.

**Results:**

No group difference was found for working memory capacity, but individuals with SCZ showed diminished trial-by-trial recall accuracy, showed reduced pupil dilation across all task load conditions, and reported higher perceived task demands.

**Conclusion:**

Results indicate reduced effort investment in patients with SCZ, but it remains unclear to what extent this alone could explain the lower recall performance. The lack of a direct link between objective and subjective measures of effort further suggests that both may assess different facets of effort. This has important implications for clinical and research settings that rely on the reliability of neuropsychological test results when assessing cognitive capacity in this patient group.

## Introduction

Working memory deficits are commonly reported in persons with schizophrenia (SCZ; e.g., Horan et al., 2008; Ventura et al., 2009; Freeman et al., 2014) and have been explained by a lack of processing resources (Nuechterlein and Dawson, 1984; Granholm et al., 1997). However, persons with SCZ, particularly when negative symptoms are prevalent, seem to be less willing to engage with physically (Gold et al., 2013; Barch et al., 2014; Bergé et al., 2018) or cognitively effortful tasks (Wolf et al., 2014; Gold et al., 2015; Culbreth et al., 2016; Reddy et al., 2018; Chang et al., 2019) and, when engaged, tend to exert less effort during task performance (Gorissen et al., 2005; Granholm et al., 2006, 2016). Accordingly, diminished performance on cognitive tests of persons with SCZ might be explained not only by real cognitive impairments or limited resources but also by reduced invested effort (Gorissen et al., 2005). This has important implications for neuropsychological test situations in both clinical and research applications and led some authors to call for a combined assessment of neuropsychological performance and mental effort in persons with SCZ (Gorissen et al., 2005).

Mental effort has been described as the mediating processes between the theoretically achievable level of performance determined by task demands and cognitive capacity, and the actual level of performance achieved (Shenhav et al., 2017). These processes are affected by both cognitive and motivational factors, including personal goals, incentives, personality, and metacognitive knowledge (Fisher and Ford, 1998; Paas et al., 2005). Effort is inherently aversive and costly, as it requires the mobilization of energy (Gaillard, 1993; Fairclough and Houston, 2004; Shenhav et al., 2017). Hence, reduced effort exertion in persons with SCZ may be related to an overestimation of those (internal) costs (Gold et al., 2015; Shenhav et al., 2017) and could be related to a decreased tolerance of strain (van den Bosch and Rombouts, 1997). Measuring mental effort accurately has proven difficult. Studies investigating the willingness to exert effort often quantify this as choosing hard (high task demand) over easy tasks (low task demand) in favor of a larger monetary reward. Results may thus be confounded by subjective evaluation of monetary reward (see, e.g., Culbreth et al., 2016; Chang et al., 2019). In contrast, during standard neuropsychological assessments, no explicit external rewards are available, and patients usually cannot choose between hard and easy tasks. Measuring actual effort exertion in these contexts must therefore rely on different and more task-independent measures, for example, post-assessment self-reports (Moritz et al., 2017a). A more objective marker of mental effort exertion is pupil dilation during task performance (Granholm et al., 2016; van der Wel and van Steenbergen, 2018). The assumption that pupil dilation reflects effort allocation rests on the observation of positive correlations between pupil dilation and performance (Van Der Meer et al., 2010; Rondeel et al., 2015). Accordingly, smaller task-related pupil responses in persons with SCZ have been interpreted as an indication of reduced mental effort in SCZ and were found to be related to the severity of negative symptoms and defeatist attitudes (Granholm et al., 2006, 2016). Surprisingly, only a few studies investigated to what extent this objective measure of mental effort converges with self-reports of invested effort and motivation in these samples. Moreover, the role of subjectively perceived task demands and experienced strain remains unexplored, despite its likely detrimental role in effort investment (van den Bosch and Rombouts, 1997; Gold et al., 2015).

The current study aimed to investigate the relationship between working memory capacity, recall accuracy, pupil dilation, and subjective measurements of perceived task demands and motivated effort in a sample of participants with SCZ as compared to a sample without any psychiatric diagnosis. Participants with SCZ were expected to show smaller working memory capacity, recall accuracy, and pupil dilation as compared to participants without any psychiatric disorder across conditions of differing task demands. Further, patients were hypothesized to report higher strain caused by the task demands overall in combination with lower motivated effort. The self-report measures of strain and motivated effort were expected to correlate with the severity of negative symptoms.

## Materials and Methods

Inpatients and outpatients with a diagnosis from the SCZ spectrum were contacted directly and through the distribution of leaflets at the Department of Psychiatry and Psychotherapy of the University Medical Center Hamburg-Eppendorf (UKE), Germany. Healthy control participants were recruited through leaflets and posts on social media and student job websites. Participants had to meet the following inclusion criteria: (1) 18–65 years of age, (2) very good command of the German language, (3) IQ above 80, (4) capacity to give informed consent, (5) no substance dependence, (6) no recreational drug consumption within 1 week prior to the assessment (excluding alcohol, nicotine, and caffeine), (7) no history of neurological disorders, (8) normal or corrected-to-normal eyesight, and (9) a primary diagnosis of SCZ or schizoaffective disorder (SCZ group; DSM-V, American Psychiatric Association, 2013) or no psychiatric diagnosis at all (HC group). For all participants, written informed consent was obtained prior to the study. The study was approved by the local ethics committee of psychologists at the UKE.

This study was part of a larger project, and the total sample contained 61 participants. Only 59 of those completed the version of the digit span task and the corresponding motivation questionnaire as described here. Analyses of overall performance and questionnaires therefore rely on the data of 59 participants. For trial-wise analyses of pupil dilation and performance, another three participants were excluded due to large amounts of missing pupil data and technical difficulties during pupil recording.

### Measures

#### Visual Digit Span Task

A visual, computerized version of the digit span task was administered. All stimuli were white on gray background. A trial started with the presentation of a fixation cross (4 s). A number of digits between one and nine were then shown one after another (1 s each), with a 1-s interstimulus interval. At the end of each trial, participants had to recall the digits in the order they were presented in and manually type in their responses on a standard keyboard. To keep the task as similar as possible to the standard forward digit span subtest of the Wechsler adult intelligence scale (WAIS-IV; Wechsler, 2008), the amount of digits presented in one trial increased over time: starting off with two digits, an additional digit was added after every second trial until the maximum amount of nine digits. Thus, for each load condition between two and nine, two trials were completed. During digit presentation, pupil size was recorded at a rate of 500 Hz with a desktop-mounted infrared video-based eye tracker (Eyelink 1000, SR Research).

#### Post-assessment Questionnaire

Self-reported motivation, invested effort, and subjective task demand were assessed after completion of the digit span task. The scales were newly compiled from items of the NASA Task Load Index (N-TLX; Hart and Staveland, 1988) and an authorized adaptation of items from the Momentary Influences, Attitudes and Motivation Impact on Cognitive Performance Scale (MIAMI; Moritz et al., 2017b) to cover topics such as motivation, invested effort, perceived task difficulty, and strain. In total, 17 items were posed on a Likert scale from 1 (completely disagree) to 4 (completely agree) (example items: “The task was very easy.”; “I was very motivated.”).

#### Clinical Assessments

Clinical diagnoses (SCZ group) or the absence thereof (HC group) was confirmed with the Mini-International Neuropsychiatric Interview (MINI; Sheehan et al., 1998). Positive and negative symptoms were assessed with the Positive and Negative Symptoms Scale (PANSS; Kay et al., 1987) within the SCZ group. Since the validity of the original PANSS dimensions has been criticized, particularly with regard to the negative symptoms scale (van der Gaag et al., 2006; Khan et al., 2013), negative symptom scores were calculated both according to the original publication (subsequently PANSS-N) and according to the scoring suggestions by van der Gaag et al. (2006; subsequently PANSS-N_*vdGaag*_). As a proxy for premorbid intelligence, the German multiple choice vocabulary test (Lehrl et al., 1995) was administered.

### Analysis

For overall analyses of working memory capacity, questionnaire responses, and clinical assessments, Spearman correlations and Mann–Whitney *U*-tests were used due to violated normality assumptions. Non-parametric effect sizes are reported as Cliff’s delta *d_*C*_.* For trial-wise analyses of recall accuracy, load condition, group membership, and pupil dilation, linear mixed regression models were built hierarchically and compared with the likelihood-ratio chi-squared test. For detailed model comparison and model parameters at each step, see Supplementary Tables S1–S3. All confirmatory testing was conducted with a significance level of 0.05, using the R programming language (R version 3.5.1, R Core Team, 2018).

#### Pupil Dilation Preprocessing

Eye blinks and artifacts were detected with a custom-built filter based on the pupil signal’s velocity and removed through cubic-spline interpolation (Mathôt et al., 2018). The signal was then smoothed with a 3-Hz low-pass Butterworth filter, and periods of missing and aberrant data spanning more than 1000 consecutive milliseconds were treated as NA. Baseline pupil size for every trial was calculated as the mean pupil size of the 200 ms prior to the first digit. Percentage change in pupil size from baseline was then calculated for each sample of the trial. Baseline-corrected pupil dilation at each digit was then calculated by averaging the signal across the 1-s period after digit onset. Consistent with Granholm et al. (2016), the average pupillary response to the last digit presented in each trial was the main variable of interest. Only trials with less than 25% of missing data and where less than 50% of the signal used to calculate this main variable had been interpolated were submitted to subsequent analyses.

## Results

There were no significant group differences in any of the demographic variables or premorbid intelligence (see Table 1).

**TABLE 1:** Sample demographics per group (total sample size = 59).

	SCZ (*n* = 29)			HC (*n* = 30)			*P*
		
	*n*	*M* (*SD*)	*Md* (*IQR*)	*n*	*M* (*SD*)	Md (*IQR*)	
Gender (m/f)	14/15			13/17			0.90
Education (“1”/“2”/“3”)	1/2/26			1/5/24			0.51
Age		47.55 (11.66)	51 (15)		45.80 (11.64)	47 (16.75)	0.57
WST		33.52 (3.54)	34 (4)		32.37 (4.55)	34 (6.25)	0.28
PANSS							
Positive Scale		12.07 (4.17)	11 (6)				
Negative Scale		10.41 (3.12)	10 (4)				
Negative Scale_*vdGaag*_		12.59 (4.21)	12 (4)				
Total score		49.79 (14.24)	45 (15)				
Time since onset		19.38 (12.14)	18 (14)				
Inpatients/outpatients	5/24						

*Sample sizes (*n*), counts, means (*M*; with standard deviations *SD*), and medians (*Md*; with inter-quartile ranges *IQR*) are displayed. Education was recorded in German school system categories corresponding to completion of 1 = secondary school I (up to age 15), 2 = secondary school II (up to age 16), 3 = sixth form college (up to age 19). WST, German vocabulary test. Negative Scale_vdGaag_, negative symptom scoring according to van der Gaag et al. (2006). *P*-values for group comparisons are provided for the demographical variables gender and education (chi-squared tests) as well as age and the WST scores (*T*-test).*

The SCZ group consisted of five inpatients and 24 outpatients. Thereof, 24 participants reported taking antipsychotic medications (83%; first generation: 1; second generation: 19; both first and second generations: 4). The mean percentage of the clinically recommended maximum dosage (Kane et al., 2003) was 60.94 (*SD* = 78.84). One participant took additional anticholinergic and 11 (38%) took other psychotropic drugs.

An exploratory factor analysis with varimax rotation revealed two subscales of the post-assessment questionnaire. The first one reflected perceived task demands and to what extent participants felt challenged and stressed (including items such as “In my opinion, the task was very difficult.” and “I felt very stressed.”). This scale included seven items and was labeled “ease” due to its reverse coding (i.e., lower values reflect higher experienced task demands). The possible score range was 7–28, and Cronbach’s alpha was 0.82. The second scale reflected self-reported motivation and invested effort (including items such as “I was very motivated.” and “I put in a lot of effort and gave it my best shot.”). This scale encompassed eight items and was labeled “motivated effort” to distinguish it from effort driven by task demands (for full scales, see Supplementary Material). The possible score range was 8 to 32, and Cronbach’s alpha was 0.81.

### Overall Analyses: Maximum Digit Span and Correlation With Questionnaire Scales

General working memory capacity was assessed as the maximum number of correctly recalled digits in a row in the task overall, independent of load condition. The SCZ and the HC group only differed at a statistical trend (*Md*_*SCZ*_ = 6, *Md*_*HC*_ = 7; *W* = 551.1, *p* = 0.07, *d_*C*_* = 0.27). Both groups reported similar motivated effort (*Md*_*SCZ*_ = 25, *Md*_*HC*_ = 28; *W* = 541.5, *p* = 0.11, *d_*C*_* = 0.24). However, participants with SCZ reported smaller values for ease, i.e., they felt more challenged and strained by the task (*Md*_*SCZ*_ = 16, *Md*_*HC*_ = 19; *W* = 617.5, *p* = 0.01, *d*_*C*_ = 0.42).

There was a positive relationship between reported ease and maximum digit span across the whole sample (ρ = 0.26, *p* = 0.04) but no relationship between motivated effort and maximum digit span (ρ = 0.21, *p* = 0.12). Within the SCZ group, negative symptoms correlated neither with maximum digit span (PANSS-N: ρ = 0.03, *p* = 0.90; PANSS-N_*vdGaag*_: ρ = 0.30, *p* = 0.13), ease (PANSS-N: ρ = 0.11, *p* = 0.57; PANSS-N_*vdGaag*_:ρ = −0.03, *p* = 0.87), nor motivated effort (PANSS-N: ρ = 0.03, *p* = 0.89; PANSS-N_*vdGaag*_: ρ = 0.05, *p* = 0.80). Ease and motivated effort were moderately correlated (ρ = 0.34, *p* < 0.01).

As anticholinergic agents can have detrimental effects on cognitive functions like working memory (Spohn and Strauss, 1989; Minzenberg et al., 2004) and affect pupil size (Naicker et al., 2016), benztropine mesylate equivalents, where available, were used to assess the anticholinergic load induced by each participant’s daily dosage of the prescribed antipsychotics (Minzenberg et al., 2004). There was no difference in maximum digit span (*W* = 103, *p* = 0.98) or pupil dilation at the four-digit load condition, i.e., the load condition equivalent to the minimum digit span achieved in this sample (*W* = 69, *p* = 0.69), between participants who received an antipsychotic with a known anticholinergic effect (*Md*_*digit span*_ = 6, *Md*_*pupil*_ = 2.54, *n* = 16) and those who did not (*Md*_*digit span*_ = 6, *Md*_*pupil*_ = 1.89, *n* = 13). Anticholinergic load was correlated neither with maximum digit span (ρ = 0.26, *p* = 0.27, *n* = 20) nor with average pupil dilation at the four-digit load condition (ρ = 0.15, *p* = 0.59, *n* = 16). Similarly, the percentage of maximum dosage of all antipsychotics was not related to the maximum digit span (ρ = 0.11, *p* = 0.63, *n* = 23) or average pupil dilation at the four-digit load condition (ρ = −0.10, *p* = 0.67, *n* = 19).

### Trial-Wise Analyses: Recall Accuracy

Trial-wise recall accuracy was measured as the percentage of digits recalled in the correct order on a given trial until the first error was made. To illustrate, within a load condition of eight digits, recall accuracy would be 50% if the first four digits were remembered correctly, but digits from the fifth digit onward were reported in an incorrect order. As seen in Table 2, average recall accuracy per load condition expectedly decreased with increasing load. This was confirmed by linear mixed regressions, which revealed main effects of load, χ^2^(1) = 313.32, *p* < 0.001, and group, χ^2^(1) = 4.94, *p* = 0.03, on recall accuracy, while the interaction between load and group was not significant, χ^2^(1) = 2.23, *p* = 0.14. In the winning model with only the two main effects, recall decreased as load increased, *b* = −9.89, *t* = −22.11, *p* < 0.001, and was lower in the SCZ group as compared to the HC group, *b* = −6.56, *t* = −2.26, *p* = 0.03.

**TABLE 2:** Average percentage of items recalled in correct order per load condition for each group (*N* = 56).

Load	SCZ (*n* = 27)		HC (*n* = 29)	
		
	*M* (*SD*)	*Md* (*IQR*)	*M* (*SD*)	Md (*IQR*)
2	100 (0)	100 (0)	100 (0)	100 (0)
3	97.9 (14.6)	100 (0)	100 (0)	100 (0)
4	94.3 (22.1)	100 (0)	98.7 (10.0)	100 (0)
5	90.9 (23.0)	100 (0)	91.6 (22.7)	100 (0)
6	67.1 (36.6)	83.3 (66.7)	81.2 (29.0)	100 (33.3)
7	49.3 (38.0)	35.7 (85.7)	62.5 (37.6)	71.4 (85.7)
8	42.3 (35.0)	25 (62.5)	47.5 (35.2)	37.5 (50)
9	35.1 (36.1)	22.2 (55.6)	43.5 (35.1)	38.9 (58.3)

*Means (*M*; with standard deviations *SD*) and medians (*Md*; with inter-quartile ranges *IQR*) are displayed. Trials with NA entries for pupil dilation excluded per subject for comparability with regression models.*

### Trial-Wise Analyses: Pupil Dilation

**FIGURE 1:**
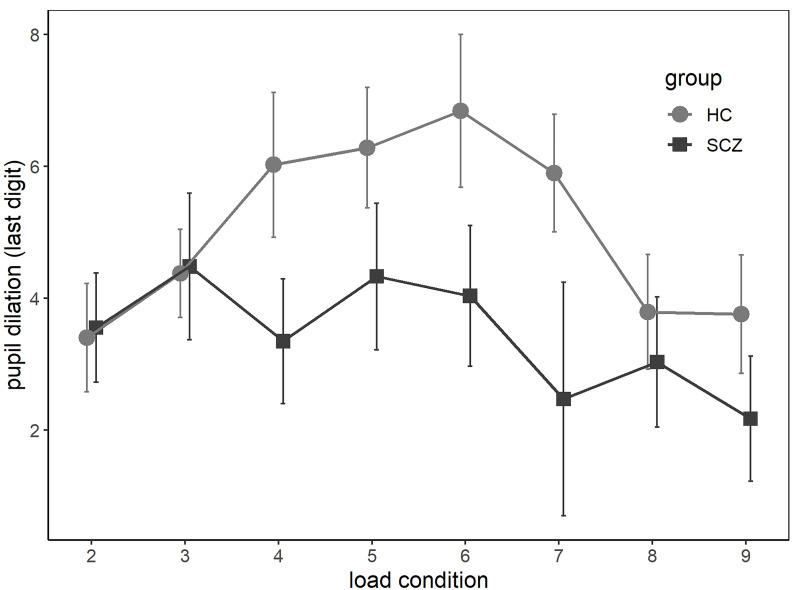
Average baseline-corrected pupil dilation at the last digit of each load condition (2–9) for participants with diagnosis from the schizophrenia spectrum (SCZ group) and without (HC group). Error bars reflect standard errors of the mean.

As seen in Figure 1, in the HC group, trial-wise pupil dilation to the last digit increased with increasing processing load before it reached asymptote and decreased in higher load conditions. In contrast, this inverse U-shaped relationship was less prevalent in the SCZ group, and pupil dilation was smaller across almost all load conditions. These observations were confirmed by linear mixed regressions. Given the observed inverse U-shaped relationship between load and pupil dilation, both linear and quadratic load terms were tested as predictors. There was no significant effect for the linear load term, χ^2^(1) = 0.95, *p* = 0.33; the reverse was true for the quadratic one, χ^2^(1) = 18.50, *p* < 0.001. There was a significant main effect of group, χ^2^(1) = 4.07, *p* = 0.04. The interaction between load and group was not significant, χ^2^(1) = 1.05, *p* = 0.31, but the interaction between quadratic load and group indicated a trend, χ^2^(1) = 2.89, *p* = 0.09. In the winning model, which included the main effects only, both the linear and quadratic load terms were significantly related to pupil dilation, linear: *b* = 2.08, *t* = 4.06, *p* = < 0.001; quadratic: *b* = -0.20, *t* = -4.31, *p* = < 0.001. Further, participants with SCZ showed generally smaller pupil dilation across load conditions, *b* = -1.77, *t* = -2.04, *p* = 0.046. Notably, there was no group difference in baseline pupil size across all trials, χ^2^(1) = 2.37, *p* = 0.12.

### Trial-Wise Analysis: Can Pupil Dilation at Last Digit Predict Recall Accuracy?

In another linear mixed regression analysis, the final model from Section “Trial-Wise Analyses: Recall Accuracy” was extended to establish if pupil dilation could predict variance in performance above and beyond the amount explained by load condition and group membership. Adding pupil dilation to the model indeed improved it significantly, χ^2^(1) = 4.58, *p* = 0.03. In this model, coefficients for load and group were consistent with the results of Section “Trial-Wise Analyses: Recall Accuracy,” with performance decreasing as load increased, *b* = -9.86, *t* = -22.22, *p* < 0.001, and being lower in the SCZ as opposed to the HC group, *b* = -6.00, *t* = -2.04, *p* = 0.046. In line with an interpretation of pupil size as a measure of invested mental effort, larger pupil dilation predicted better performance, *b* = 0.32, *t* = 2.15, *p* = 0.03.

To test if this relationship was similar for all load and group conditions, interaction effects were added. The interaction term of load and group was not significant, χ^2^(1) = 1.63, *p* = 0.20, and therefore excluded from further models. However, the interactions between load and pupil dilation, χ^2^(1) = 5.14, *p* = 0.02, and between group and pupil dilation, χ^2^(1) = 4.59, *p* = 0.03, improved the model significantly. The final model therefore included load, group, pupil dilation, and the interactions between load and pupil, as well as group and pupil. Here, recall accuracy decreased with increasing load, *b* = -10.34, *t* = -20.62, *p* < 0.001, but in the presence of the interaction terms, there was no significant main effect for group, *b* = -3.05, *t* = -0.99, *p* = 0.33, or pupil dilation, *b* = -0.05, *t* = -0.11, *p* = 0.91. There was a meaningful trend for the interaction between load and pupil dilation, *b* = 0.12, *t* = 1.86, *p* = 0.06, indicating that the detrimental effect of load on performance was smaller on trials with larger pupil responses. Further, the interaction between group and pupil dilation was significant, *b* = -0.65, *t* = -2.16, *p* = 0.03, suggesting that pupil dilation was less predictive of performance in the SCZ as compared to the HC group.

### Overall Analysis: Pupil Dilation and Subjective Effort in Max Span Condition

Linear mixed regression analyses for pupil dilation in the four-digit trials were conducted to explore the relationship between pupil dilation and the self-report questionnaire. This load condition was chosen because four was the minimum working memory capacity within the whole sample. Thus, a negative relationship between pupil dilation and maximum digit span within this condition would be expected as participants with more available cognitive resources would need to invest less effort (relative to their cognitive capacity) than persons with fewer resources. Adding self-reported motivated effort and perceived ease to the model while controlling for capacity and group would then give an indication to what extent pupil dilation is affected by motivational factors in addition. Since motivated effort and ease were correlated, two separate models were built. In the motivated effort model, only the group effect that had already being observed across all load conditions achieved marginal significance (*b* = -3.02, *t* = -1.97, *p* = 0.05, *n* = 54), but no effect of maximum digit span (*b* = -0.48, *t* = -0.90, *p* = 0.37, *n* = 54) or motivated effort (*b* = 0.04, *t* = 0.20, *p* = 0.84, *n* = 54) was found. Results from the ease model were similar, with no effects for maximum digit span (*b* = -0.36, *t* = -0.68, *p* = 0.50, *n* = 54) or ease (*b* = -0.26, *t* = -1.19, *p* = 0.24, *n* = 54), but smaller pupil dilation in the SCZ group (*b* = -3.49, *t* = -2.26, *p* = 0.03, *n* = 54). Within the SCZ group, the average pupil dilation in the four-digit trials was not related to negative symptoms (PANSS-N:ρ = 0.01, *p* = 0.95, *n* = 25; PANSS-N_*vdGaag*_:ρ = -0.09, *p* = 0.68).

## Discussion

This study investigated the relationship between performance in a working memory task, self-reported motivated effort and ease, and objective effort allocation as indexed by pupil dilation in individuals with a clinical diagnosis from the SCZ spectrum (SCZ group) and individuals with no psychiatric disorder (HC group).

While there was no significant group difference in working memory capacity measured as maximum digit span, the SCZ group showed decreased recall accuracy on a trial-by-trial basis. The absence of a significant difference in maximum digit span may seem surprising, as working memory deficits in SCZ are well established. However, not all studies using the digit span task have replicated this finding (Park and Holzman, 1992; Franke et al., 1993). In the current study, participants had multiple opportunities to demonstrate their general working memory capacity, as performance in all trials were considered when assessing maximum digit span. In contrast, trial-by-trial assessment of recall accuracy may have been more sensitive to momentary fluctuations in attention, which in turn might be affected by motivation (Engelmann et al., 2009). Given similar general capacity in both groups, at first glance, the differences in trial-wise performance seem more likely to have been caused by reduced effort rather than by a general lack of cognitive resources. In line with this, pupil dilation was reduced in the SCZ group across all load conditions, suggesting that participants with SCZ indeed invested less effort while doing the task. The inverse U-shaped relationship between load and pupil dilation was present across groups, though more prominent in the HC group, and can be interpreted as a detachment from the task at hand as task demands exceed available cognitive resources and thus decreasing expectations of success (Granholm et al., 2016). While some studies found group differences in pupil dilation only for high task demands (Granholm et al., 1997, 2006), others have reported differences across all demands, similar to the findings of this study (Granholm et al., 2016). Such discrepancies are likely the result of methodological differences and categorization of high and low demands. While the interaction effect between load and group on pupil dilation did not reach significance, the descriptive results suggest that pupil dilation was actually similar in trials where task load was below four digits (see Figure 1).

The interpretation of trial-wise pupil dilation as a measure of effort was supported by its positive relationship with trial-wise recall accuracy in a basic linear mixed regression model. In the regression model with interaction terms, recall accuracy of participants with larger pupil responses declined less as task load increased. Thus, increased task load can be compensated with an increase in invested effort. Nevertheless, the significant interaction between pupil dilation and group suggested that the positive relationship between pupil dilation and performance was smaller, if not absent, in the SCZ group. This makes it difficult to conclude if decreased trial-by-trial performance in this group can truly be attributed to less effort and proposes the role of additional explanatory factors. Interestingly, participants with SCZ reported feeling more challenged and stressed by the task, and this feeling of strain was correlated with maximum digit span and with motivated effort across the entire sample. On the one hand, it is likely that limited cognitive capacity leads to higher perceived task demands and strain. On the other hand, the cognitive resources available might not be exploited fully in situations where the task environment induces stress, which in turn may lead to an increase in perceived strain (Fairclough and Houston, 2004). Momentary sensitivity to stress has, in fact, been found to negatively affect cognitive functioning in SCZ (Morrens et al., 2007). Similarly, a generally reduced tolerance of strain in persons with SCZ could potentially explain the pattern of findings including heightened self-reported strain, smaller pupil dilation, and impaired recall accuracy across all load conditions (van den Bosch and Rombouts, 1997). This interpretation fits also well with the idea that persons with SCZ may invest less effort as a consequence of an overestimation of the costs associated with it (Gold et al., 2015; Shenhav et al., 2017). However, self-reported ease (i.e., reversed strain) did not predict pupil dilation in the four-digit trials and neither did self-reported motivated effort. Further, self-reported effort did not differ between groups, conflicting with the finding of smaller pupil dilation in SCZ across the task. This indicates little convergence between subjective and objective measures of effort, which may be linked in part to the way both constructs were measured (trial wise vs. after task completion) and to the fact that self-reports can be biased by lack of retrospective insight as well as social desirability.

None of our variables of interest correlated with negative symptom severity. This may seem unexpected, as previous studies have demonstrated a negative relationship between negative symptom severity and effort investment (e.g., Gorissen et al., 2005; Wolf et al., 2014) or that effort investment was predominantly impaired in subgroups scoring high on negative symptoms (Granholm et al., 2006; Bergé et al., 2018). However, other findings indicate that the relationship between effort investment and negative symptoms may, in fact, be non-linear and moderated by other factors, such as defeatist attitudes (Granholm et al., 2016; Reddy et al., 2018). Given the small sample size and the rather low average negative symptom score of the patient sample, no subgroups of high- and low-scoring patients were compared in the current study. The low scores were likely related to the large percentage of outpatients who tend to express fewer negative and other symptoms (e.g., Kasckow et al., 2001). Note that inconsistencies in findings regarding negative symptoms can further be related to the fact that measurement instruments differ across studies. The PANSS, which was chosen here, has received criticism for not reflecting the latest research results on negative symptoms (Kumari et al., 2017), which poses a limitation on the interpretability of the findings.

Further limitations of the study include the rather small sample sizes (particularly for the analyses including medication variables), the fact that medication was self-reported, the heterogeneity of the sample in terms of mixing in- and outpatients and including participants with schizoaffective disorders, as well as the possibility that matching groups by level of education may have contributed to the selection of an atypical, high-achieving group of persons with SCZ (Resnick, 1992). All of these factors may explain why some results from previous studies could not be replicated. The sample may have also been biased by the large proportion of chronically ill patients who, in turn, have been exposed to antipsychotic medication for long periods of their lives.

One potential limitation of the design is the fact that task load conditions were not randomized to ensure comparability with the standard version of the digit span subtest from the WAIS-IV (Wechsler, 2008). However, depletion or fatigue effects (Hagger et al., 2010) cannot account for the consistently smaller pupil dilation in SCZ across all load conditions. Another limitation is that subjective effort was only assessed after task completion with scales that have not been externally validated, although they were derived from well-validated measures.

Taken together, the findings of this study demonstrate once again the complex relationships between performance, effort, cognitive resources, and task demands. The results involving pupil dilation suggest that, in cognitive tasks, participants with SCZ might indeed exert less mental effort. However, it remains unclear to what degree this accounts for impaired momentary performance in this sample and to what extent this is linked to the higher perceived strain imposed by task demands. To accurately judge the outcome of clinical or research-related neuropsychological assessments, these and other motivational factors have to be taken into account. Importantly, the lack of convergence between subjective and objective measures of effort might indicate that both objective and subjective measures can complement each other in unique ways and should thus be both considered for applications in this context.

## Data Availability Statement

All datasets generated for this study are included in the article/Supplementary Material. Data as well as task and questionnaire material are available in an Open Science Framework repository: 10.17605/OSF.IO/GCH97.

## Ethics Statement

The studies involving human participants were reviewed and approved by the ethics committee of psychologists at the University Medical Center Hamburg-Eppendorf, Hamburg, Germany. The patients/participants provided their written informed consent to participate in this study.

## Author Contributions

All authors contributed to the article and approved the submitted version. IK, SM, and GP designed the study and edited the manuscript. IK collected and analyzed the data and wrote the manuscript.

## Conflict of Interest

The authors declare that the research was conducted in the absence of any commercial or financial relationships that could be construed as a potential conflict of interest.
