# Supplementary material for: Objective Versus Subjective Effort in Schizophrenia
Source: Front Psychol. 2020 Jul 9;11:1469. doi: 10.3389/fpsyg.2020.01469 (PMC7365134; doi:10.3389/fpsyg.2020.01469)
Supplement: Supplementary file 1 [file Table_1.docx]

# Supplementary material

All trial-wise linear mixed regression models were built hierarchically using the *nlme* R package (Pinheiro, Bates, DebRoy, & Sarkar, 2013), meaning predictors were added one after another and model fit was tested using likelihood-ratio chi-squared test at each step of the process. The following tables show model parameters for each of these steps as well as the corresponding pseudo-R^2^. Those coefficients of determination were calculated using the *piecewiseSEM* R package (Lefcheck, 2016) and specify both the proportion of variance explained by the fixed effect(s) alone (Marginal *R^2^*; *R^2^m*) as well as the proportion of variance explained by both the fixed and random effects (conditional *R^2^*; *R^2^c*) as described in (Nakagawa & Schielzeth, 2013).

## Trial-wise analyses: recall accuracy

Table S1

*Summary of hierarchical linear mixed regression*

*analysis for variables predicting recall accuracy*

*(percentage of digits recalled in the correct order)*

*per trial*

| Variable | | *b* | *t* | *p* | *R^2^_M_* | *R^2^_C_* |
| --- | --- | --- | --- | --- | --- | --- |
| *Step 1* |  |  |  |  | 0.400 | 0.562 |
|  | load | -9.89 | -22.12 | <.001 |  |  |
| ***Step 2*** |  |  |  |  | **0.408** | **0.563** |
|  | load | -9.89 | -22.11 | <.001 |  |  |
|  | group | -6.56 | -2.26 | 0.03 |  |  |
| *Step 3* |  |  |  |  | 0.410 | 0.564 |
|  | load | -9.29 | -15.37 | <.001 |  |  |
|  | group | 0.77 | 0.14 | 0.89 |  |  |
|  | load x group | -1.34 | -1.49 | 0.14 |  |  |

*Notes:* Trials with NA entries for pupil dilation excluded per subject

for comparability with pupil dilation models. Group is coded as 0

for HC (baseline) and 1 for SCZ. Winning model in **bold**.

## Trial-wise analyses: pupil dilation

Table S2

*Summary of hierarchical linear mixed regression*

*analysis for variables predicting pupil dilation at last*

*digit per trial*

| Variable | | *b* | *t* | *p* | *R^2^_M_* | *R^2^_C_* |
| --- | --- | --- | --- | --- | --- | --- |
| *Step 1* | |  |  |  | 0.001 | 0.191 |
|  | load | -0.09 | -0.98 | 0.33 |  |  |
| *Step 2* |  |  |  |  | 0.020 | 0.215 |
|  | load | 2.09 | 4.08 | <.001 |  |  |
|  | load^2 | -0.20 | -4.32 | <.001 |  |  |
| ***Step 3*** |  |  |  |  | **0.037** | **0.215** |
|  | load | 2.08 | 4.06 | <.001 |  |  |
|  | load^2 | -0.20 | -4.31 | <.001 |  |  |
|  | group | -1.77 | -2.04 | 0.046 |  |  |
| *Step 4* |  |  |  |  | 0.038 | 0.216 |
|  | load | 2.17 | 4.17 | <.001 |  |  |
|  | load^2 | -0.20 | -4.31 | <.001 |  |  |
|  | group | -0.74 | -0.56 | 0.58 |  |  |
|  | load x group | -0.19 | -1.02 | 0.31 |  |  |
| *Step 5* |  |  |  |  | 0.041 | 0.218 |
|  | load | 2.93 | 4.27 | <.001 |  |  |
|  | load^2 | -0.27 | -4.35 | <.001 |  |  |
|  | group | 3.16 | 1.19 | 0.24 |  |  |
|  | load x group | -1.91 | -1.85 | 0.07 |  |  |
|  | load^2 x group | 0.16 | 1.69 | 0.09 |  |  |

*Notes:* Trials with NA entries for pupil dilation excluded per subject.

Group is coded as 0 for HC (baseline) and 1 for SCZ. Winning model

in **bold**.

## Trial-wise analyses: recall accuracy & pupil dilation

Table S3

*Summary of hierarchical linear mixed regression analysis*

*for pupil dilation and other variables predicting recall*

*accuracy (percentage of digits recalled in the correct*

*order) per trial*

| Variable |  | *b* | *t* | *p* | *R^2^_M_* | *R^2^_C_* |
| --- | --- | --- | --- | --- | --- | --- |
| *Step 1* |  |  |  |  | 0.400 | 0.562 |
|  | load | -9.89 | -22.12 | <.001 |  |  |
| *Step 2* |  |  |  |  | 0.408 | 0.563 |
|  | load | -9.89 | -22.11 | <.001 |  |  |
|  | group | -6.56 | -2.26 | 0.03 |  |  |
| *Step 3* |  |  |  |  | 0.411 | 0.561 |
|  | load | -9.86 | -22.22 | <.001 |  |  |
|  | group | -6.00 | -2.04 | 0.046 |  |  |
|  | PD | 0.32 | 2.15 | 0.03 |  |  |
| *Step 4a* |  |  |  |  | 0.413 | 0.561 |
|  | load | -9.29 | -15.50 | <.001 |  |  |
|  | group | 0.94 | 0.17 | 0.87 |  |  |
|  | PD | 0.31 | 2.11 | 0.04 |  |  |
|  | load x group | -1.27 | -1.42 | 0.16 |  |  |
| *Step 4b* |  |  |  |  | 0.415 | 0.563 |
|  | load | -10.40 | -20.25 | <.001 |  |  |
|  | group | -5.79 | -1.98 | 0.053 |  |  |
|  | PD | -0.49 | -1.27 | 0.21 |  |  |
|  | load x PD | 0.15 | 2.26 | 0.02 |  |  |
| ***Step 5*** |  |  |  |  | **0.420** | **0.560** |
|  | load | -10.34 | -20.62 | <.001 |  |  |
|  | group | -3.05 | -0.99 | 0.33 |  |  |
|  | PD | -0.05 | -0.11 | 0.91 |  |  |
|  | load x PD | 0.12 | 1.86 | 0.06 |  |  |
|  | group x PD | -0.65 | -2.16 | 0.03 |  |  |

*Notes:* PD = pupil dilation. Trials with NA entries for PD excluded per

subject. Group is coded as 0 for HC (baseline) and 1 for SCZ. Winning

model in **bold**.

# References (suppl.)

Lefcheck, J. S. (2016). piecewiseSEM: Piecewise structural equation modelling in r for ecology, evolution, and systematics. *Methods in Ecology and Evolution*, *7*(5), 573-579.

Nakagawa, S., & Schielzeth, H. (2013). A general and simple method for obtaining R2 from generalized linear mixed‐effects models. *Methods in ecology and evolution*, *4*(2), 133-142.

Pinheiro, J., Bates, D., DebRoy, S., Sarkar, D., & Team, R. C. (2013). nlme: Linear and nonlinear mixed effects models. R package version, 3(1), 111.

# Post-assessment questionnaire – items

Note that items are translated from German.

Response scale from 1 (completely disagree) to 4 (completely agree)

(R) = reversed coding of item

Subscale I: Ease *(reversed coding of strain, subjectively experienced task demands)*

- The task was very strenuous. (R)
- The task was very easy.
- I found the task very difficult. (R)
- I had to work very hard to achieve what I accomplished here. (R)
- I was very stressed. (R)
- I was very insecure. (R)
- I put a lot of pressure on myself. (R)

Subscale II: Motivated effort

- I tried hard and I did my best.
- I enjoyed this task.
- The task was too long and in the end, I did not feel like it anymore. (R)
- I was unmotivated and therefore did not perform optimally. (R)
- I was very motivated.
- I believe I was successful in implementing what was required of me.
- I was very focused on the task.
- I have tried very hard.
